# Evaluation of the ‘H2NOE Water Schools’ programme to promote water consumption in elementary schoolchildren: a non-randomised controlled cluster trial

**DOI:** 10.1017/S1368980021003438

**Published:** 2022-01

**Authors:** Ursula Griebler, Viktoria Titscher, Michael Weber, Lisa Affengruber

**Affiliations:** 1 Department for Evidence-based Medicine and Evaluation, Danube University Krems, Dr. Karl-Dorrek-Straße 30, 3500 Krems, Austria; 2 Department of Biomedical Imaging and Image-guided Therapy, Medical University of Vienna, Währinger Gürtel, Vienna, Austria

**Keywords:** School health promotion, Water consumption, Non-randomised controlled study

## Abstract

**Objective:**

This study evaluated a simple environmental intervention called ‘Water Schools’ in Lower Austria providing free refillable water bottles and educational material.

**Design:**

Non-randomised controlled cluster trial with three measurements: at baseline (T0), after the intervention at 9 months (T1) and after 1-year follow-up (T2).

**Setting:**

Half-day elementary schools in Lower Austria (Austria).

**Participants:**

Third-grade pupils from twenty-two schools in the intervention group (IG) and thirty-two schools in the control group (CG) participated in the study. Data were analysed for 569 to 598 pupils in the IG and for 545 to 613 in the CG, depending on the time of measurement.

**Results:**

The consumption of tap water increased in the IG from baseline to T1 and then decreased again at T2, but this was similar in the CG (no statistically significant difference in the time trend between the IG and CG). Similar results were seen for tap water consumption in the mornings. The proportion of children who only drank tap water on school mornings increased significantly from baseline to T1 in the IG compared to the CG (*P* = 0·020). No difference in the changes over time occurred between the groups for the proportion of pupils drinking approximately one bottle of tap water during school mornings.

**Conclusions:**

Not only the children in the IG but also those in the CG drank more tap water after 1 school year than at the beginning. The measurement of drinking habits in the CG may have been intervention enough to bring about changes or to initiate projects.

Establishing healthy dietary patterns in childhood is especially important because research suggests that dietary patterns^(1–5)^ and food preferences^(6,7)^ may track into adolescence or adulthood. In the case of sugar-sweetened beverages (SSB), a longitudinal analysis has shown that consumption of sweetened beverages increased from childhood to young adulthood^(8)^. SSB are a leading source of added calories and sugars^(9)^, and the intake of free sugars especially in the form of SSB contributes to an unhealthy diet, weight gain^(10–12)^, an increased risk of metabolic syndrome and type 2 diabetes^(13)^ and CVD^(14)^. Furthermore, dental caries is a concern, especially in children^(15–18)^. The WHO therefore strongly recommends a reduced intake of free sugars throughout the lifecourse^(19)^. Ideally, SSB should be replaced with water.

Water is essential for life, as a component for the body, and adequate fluid intake and hydration are critical for human performance and functioning^(20)^. Studies have shown that a considerable number of schoolchildren are mildly dehydrated at the beginning of the school day^(21)^, and that children’s fluid intake is inadequate as the school day progresses^(22,23)^. Availability of drinking water in school and especially in class, for example, by allowing water bottles or cups on the desk or teachers reminding children to drink during the day, has been shown to increase children’s hydration status^(21,23)^. Furthermore, water consumption has been shown to benefit cognitive performance^(24)^, short-term memory^(21)^ and both visual attention and fine motor skills in schoolchildren^(25,26)^.

The European Food Safety Authority (EFSA) defines an adequate intake of water from food and beverages as 1600 ml/d for boys and girls aged 4–8 years and 1900 ml/d for girls and 2100 ml/d for boys aged 9–13 years^(27)^. The reference values for the intake of water in the form of beverages for Germany, Austria and Switzerland are 940 ml/d for children 4–7 years old and 970 ml/d for those 7–9 years old^(28)^. However, research has shown that children between 4 and 13 years of age only reach water drinking levels between approximately 400 and 660 ml/d^(29–31)^.

Health promotion in schools is an efficient and effective way to reach a large number of young people and their families, as children spend a considerable portion of their day in school^(32)^. Schools have the potential to impact children’s behaviour by increasing the availability of healthy foods^(33)^. Furthermore, the school environment and the rules implemented in schools can affect children’s behaviour^(34)^.

Previous research on interventions to increase the water consumption among schoolchildren is inconsistent. Especially in the USA, installing drinking water sources, such as water fountains, water vessels or water bottle filling stations, has been shown to increase the water consumption of children^(35)^. Studies used different intervention strategies, mostly multi-component interventions with the provision of water bottles and educational interventions, informational material and peer agents. Some studies showed increased water consumption in the intervention groups (IG)^(36,37)^, whereas others found no difference^(38)^. However, these were small studies with only a few schools in the intervention and control arms. One large cluster-randomised controlled trial carried out in Germany combined an environmental intervention (i.e. the installation of water fountains and provision of water bottles) with educational interventions and showed an increase of 1·1 glasses of water per d between the intervention and control groups (CG)^(39)^. These studies showed that a rather simple intervention can have an effect on water consumption among schoolchildren.

In autumn 2018, the Austrian federal state of Lower Austria started a pilot programme to increase the tap water consumption of elementary schoolchildren, the ‘H2NOE Wasserschule in NÖ’ – in English, the ‘H2NOE Water Schools in Lower Austria’ programme. The aim of this study was to discern whether the programme can increase the consumption of tap water among schoolchildren.

## Methods

### Study design

This non-randomised controlled cluster trial was conducted in Austria. Due to practical reasons, we could not randomise the schools to be divided into an IG and a CG. We used the following assumptions to calculate the required sample size of our study: power 80 %, *α* 5 % (two-sided), average number of pupils per cluster: 22 ± 2, variability (sd) of consumed glasses (approximately 200 ml) between children = 0·7 glasses and a 0·3 intra-class correlation. Based on our power calculations to detect changes in water consumption, and with an assumed medium effect of a difference of 0·5 glasses/d between the IG and CG, we needed to recruit 23 school classes for each group. We had originally planned to include only one of the third-grade classes of each school. However, during the recruitment process, it turned out that there were fewer pupils per class than initially assumed, and after consultation with our statistician, we included all classes from the third grade of each school.

### Setting and participants

The study population comprised children attending third-grade elementary school (approximately 8 years old) in the federal state of Lower Austria. We chose the third grade because elementary schools in Austria last for 4 years and, thereafter, children switch to other schools and can no longer be traced. Schools were eligible to participate if they were half-day elementary schools (lessons only until before lunch) and for the IG only if they did not offer juice in the school milk programme. All participating pupils had to provide a written declaration of consent by a parent or guardian to take part in the study.

The schools in the IG were from three districts in Lower Austria (St. Pölten Land, Tulln and Melk). All elementary schools from two of these districts were contacted in June 2018 by our practice partner, who developed and delivered the programme along with experts in school health promotion, and these schools were invited to participate in the ‘H2NOE Water Schools in Lower Austria’ programme. Because we had not reached the target number of schools by the start of the 2018–2019 school year, we invited three additional schools from an adjacent district to participate in the intervention at the beginning of September 2018.

For the CG, all elementary schools from three different districts in the federal state of Lower Austria (Gänserndorf, Hollabrunn and Mistelbach) were contacted in June 2018 and invited to take part in a survey about the eating and drinking habits of children in the third grade; hence, these schools were not aware that they acted as a CG. To confirm their commitment to participate in the survey, we contacted all schools that showed an initial interest at the beginning of the school year again at the end of August/beginning of September 2018.

**Fig. 1:**
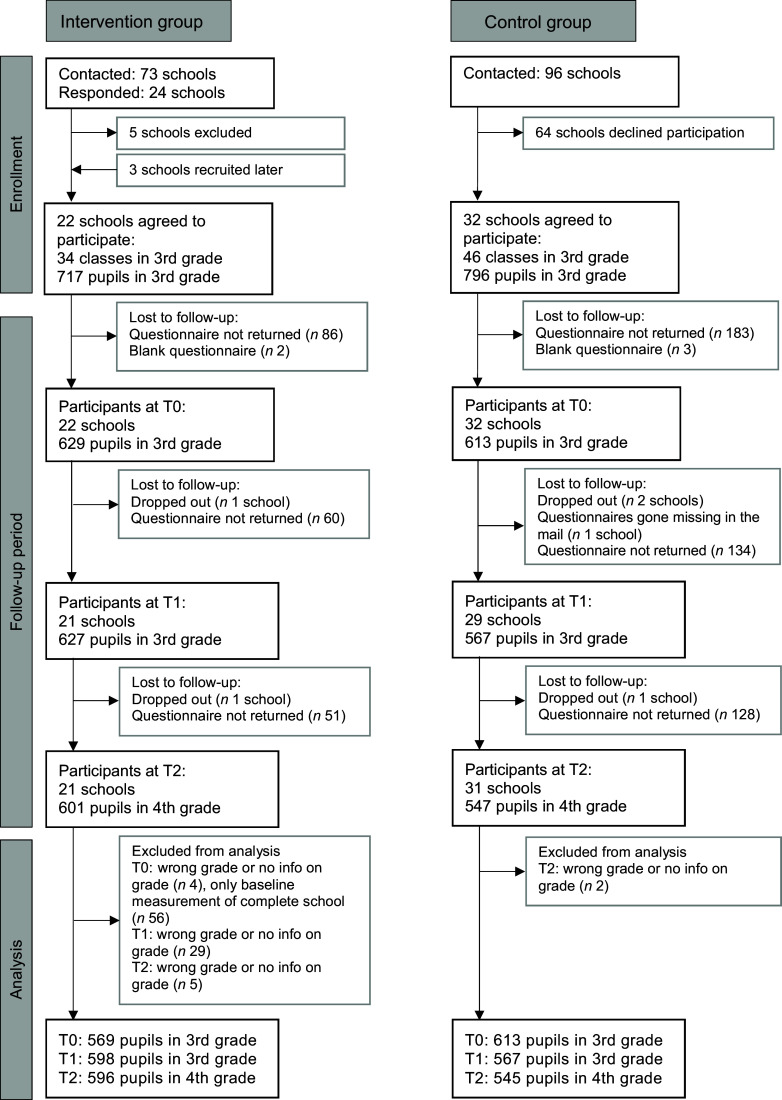
Participant and school flow in the intervention and control groups. T0, baseline; T1, after the intervention at 9 months; T2, 1-year follow-up after the intervention

Figure 1 shows the school and participant flow through the study. Twenty-one schools in the IG participated in all three measurements; one school in the IG dropped out after the baseline measurement due to time-consuming administration and questionnaire completion. In the multi-level analyses, we excluded the baseline measurements of this school. Pupils in the IG who dropped out of the study were no different than other pupils in the IG in terms of age, sex, total beverage consumption per d and juice or soft drink consumption (data not shown). However, dropouts had a higher consumption of tap water per d (5·5 glasses ± 0·4 se (*n* 50) *v*. 3·9 glasses ± 0·1 se (*n* 544), *P* = 0·041) and tap water consumption relative to total drinking volume (70·0 % ± 3·9 se (*n* 50) *v*. 48·0 % ± 1·4 se (*n* 544) *P* = 0·014) compared to other pupils in the IG.

In the CG, thirty-two schools participated in the baseline measurements in autumn 2018 (T0), twenty-nine schools took part after the intervention at the end of the school year in June 2019 (T1) because two schools did not participate and the questionnaires of one school were lost in the mail. At the 1-year follow-up in June 2020 (T2), one school did not participate (*n* 31). Because the schools that did not participate were different at different time points, we used all the data from the CG in our multi-level analyses.

In total, data were available for 1186 pupils at baseline, 1194 at T1 and 1148 at T2. The response rates for the pupil questionnaires in the participating schools were generally high, with a mean response rate of 92 % at baseline, 93 % at T1 and 94 % at T2 for the IG (range from 68 to 100 %) and slightly lower in the CG, with 79 % at baseline, 80 % at T1 and 82 % at T2 (range from 38 to 100 %).

### Intervention

In the intervention schools, each pupil and teacher received a free refillable water bottle (500 ml) made of Tritan™ copolyester, and each classroom got a free drying rack for the water bottles. In each intervention school, one information workshop was held by external nutrition experts specialising in school health promotion for all class teachers, and teachers received further educational and teaching material, for example, pre-printed posters for drinking rules or pre-prints to record the children’s fluid intake. Schools further received informational material for parents. Teachers were not instructed to give a certain number of classroom lessons dealing with the topics of water and drinking; instead, teachers decided on their own which interventions they would implement in their classroom. However, class teachers were advised to establish drinking rules along with the pupils and to complete the provided pre-printed poster for the drinking rules as well as to prompt the children to record their beverage intake on pre-printed ‘drinking passes’ at least once during the school year. In all the IG classrooms, pupils had the opportunity to fill their bottles with high-quality tap water from common handwashing basins. The school was advised to organise a whole-school event around the topics of water and water consumption for all pupils, with the aim of raising awareness among the entire school.

The intervention started with the information workshop for teachers in autumn 2018, and the intervention lasted the whole 2018–2019 school year until June 2019. However, children did not have to return the refillable water bottles after the intervention period. On the contrary, schools received further drinking bottles for the new first graders.

The schools in the CG did not receive any intervention.

### Logic model

Based on a review of the previous literature on interventions to increase the water consumption of schoolchildren, we developed a logic model together with our practice partner to refine the intervention components and to define the outputs and outcomes. The logic model was developed prior to the start of the intervention to help in specifying the intended outcomes and the possible causal pathways between the intervention components, outputs and outcomes. Logic models are often used to facilitate programme development and evaluation^(40,41)^. The main aim of the ‘H2NOE Water Schools’ intervention was that pupils exclusively drink tap water during their school day, which lasts until before lunch, reaching approximately one bottle of water (500 ml) per school day.

### Outcome measures

#### Beverage consumption

The primary outcome was a change in pupils’ tap water consumption per d and during school mornings. Changes in beverage consumption for other beverage categories were the secondary outcomes. To record the beverage consumption, we used a validated 24-h recall questionnaire developed by Muckelbauer *et al.* in 2009^(42)^ and adapted it according to their recommendations (i.e. omitting the quantity category ‘empty glass’ and the category ‘other beverages’). The questionnaire is picture-based, and children were asked to mark the number of consumed glasses of tap water, tea (fruit and herbal), mineral water, milk and chocolate milk, soft drinks (lemonade, cola and iced tea) and juice (including juice with water) for five defined time periods over the previous 24 h. The questionnaire was self-completed under teachers’ supervision at baseline (T0), after the intervention at 9 months (T1) and at the 1-year follow-up (T2) after the completion of the intervention period. Questionnaires recording a daily beverage consumption of less than two glasses or more than twenty glasses (with one glass defined as 200 ml) were classified as implausible. The secondary outcomes were the changes per d in the consumption of beverages from the other beverage categories.

For the analyses, we further calculated the following variables: tap water and total water (tap and mineral) relative to the total drinking volume (in %) and the proportion of children who only drank tap water in the morning during school and the proportion of children who drank approximately one bottle of tap water during school mornings.

We pilot-tested the entire pupil questionnaire using cognitive interviews with three children (two boys and one girl of 8 years old) at baseline (T0) and with two children (two girls of 8 and 9 years old) at T1 and made amendments accordingly. Cognitive interviews are an effective tool to identify potential problems in survey questions^(43)^. During the cognitive interviews, we asked the children to describe their thoughts when filling in the questionnaire and to indicate any difficulties in comprehending the questions or with selecting an answer.

#### Attitude of schoolchildren towards drinking

The change in pupils’ attitude towards drinking water was the second primary outcome, assessed at all three time points (T0, T1 and T2). We measured a pupil’s preference for various beverages as their agreement with various statements regarding water and SSB consumption with a five-point Likert scale (answer categories: yes, that’s right/yes, mostly/no, mostly not/no and not true/I don’t know). Furthermore, we asked pupils to choose their favourite beverage when thirsty from four predefined answer categories (tap water, mineral water, soft drinks, juice or juice with water) and one open category, where children could write down their favourite beverage when thirsty. We classified the answers as missing when more than one beverage category was selected.

#### Process evaluation parameters

As further secondary outcomes, we measured several process evaluation parameters regarding the implementation dose at T1 and T2 and regarding the implementation fidelity and reach at T1, that is, questions about the class rules for drinking, use of the water bottles and drinking pass and addressing the topic of water drinking during class. We administered online questionnaires to all teachers and headmasters in the IG and included questions in the pupils’ paper-and-pencil questionnaire (see online Supplemental file). Of the 264 teachers at the 21 participating IG schools at T1, 62 answered the questionnaire (mean response rate of 30 % per school, range 10–80 %). At T2, 74 of the 232 teachers at the 21 participating IG schools answered the questionnaire (mean response rate of 43 % per school, range 12–80 %).

Furthermore, we carried out three focus group discussions with a subset of schoolchildren and teachers to investigate the acceptance of the programme in more depth at T1. The results of the focus groups will be published elsewhere.

#### Health promotion activities during class lessons

We asked the class teachers in the IG and CG a general question about the health-promoting activities implemented in their class during the previous school year (at T0, T1 and T2). Teachers were asked to choose from a list of fifteen activities and could name further activities in an open category. The following four activities regarding water were part of the list: (1) drinking rules/drinking rituals; (2) stations run on the topic of water; (3) providing bottles/cups to drink from and (4) drinking water or a healthy choice of drinks as a topic in class.

### Statistical analyses

All analyses were performed with the statistical software package IBM SPSS Statistics for Windows Version 27.0. Tests for the baseline comparability between the IG and CG were conducted for age, sex distribution and the different categories for beverage consumption. In order to take clustered data into account, hierarchical linear mixed models (i.e. multi-level models) were used for the metric outcomes and generalised estimating equations for the categorical outcomes.

Continuous variables are presented as means and standard error and the binary and categorical data as proportions on an individual level unless stated otherwise. *P* < 0·05 was considered statistically significant.

## Results

### Study sample

At baseline, schoolchildren in the IG and CG did not differ regarding sex distribution and juice and soft drink consumption. Age was slightly higher in the IG, and the tap water consumption per d was higher in the IG than in the CG (Table 1). Due to collinearity problems, the model to test for intervention effects on the primary outcome of water consumption did not include covariates and was not corrected for baseline water consumption.

**Table 1:** Baseline characteristics for participants in the IG and CG

Characteristic*	IG			CG			*P*-value†
	*n*	Mean	se	*n*	Mean	se	
Participants	573			614			–
Schools	21			32			–
Classes	31			46			–
Participant per school							
*n*		27·3	18·2‡		19·2	15·0‡	–
Range		7–80			5–75		
Age in years	565	8·707	0·02	608	8·637	0·02	0·033
Male							
*n*	572	308		613	311		0·158
%	572	53·8		613	50·7		
Beverage consumption; glasses/d							
Water (tap)	540	3·94	0·13	591	3·14	0·11	0·003
Total water (tap and mineral)	540	4·91	0·13	591	4·31	0·12	0·286
Juice	540	1·75	0·09	591	1·67	0·08	0·642
Soft drinks	540	0·60	0·09	591	0·52	0·05	0·339
Tap water relative to total drinking volume (%)	540	48·05	1·35	591	40·89	1·28	0·777
Total water relative to total drinking volume (%)	540	58·89	1·26	591	55·55	1·23	0·725

IG, intervention group; CG, control group; sd, standard deviation; se, standard error of the mean.

* Unadjusted values on an individual level.

† *P*-values for differences between the IG and CG, with adjustment for clustering according to school and classroom.

‡ sd.

### Beverage consumption

**Fig. 2:**
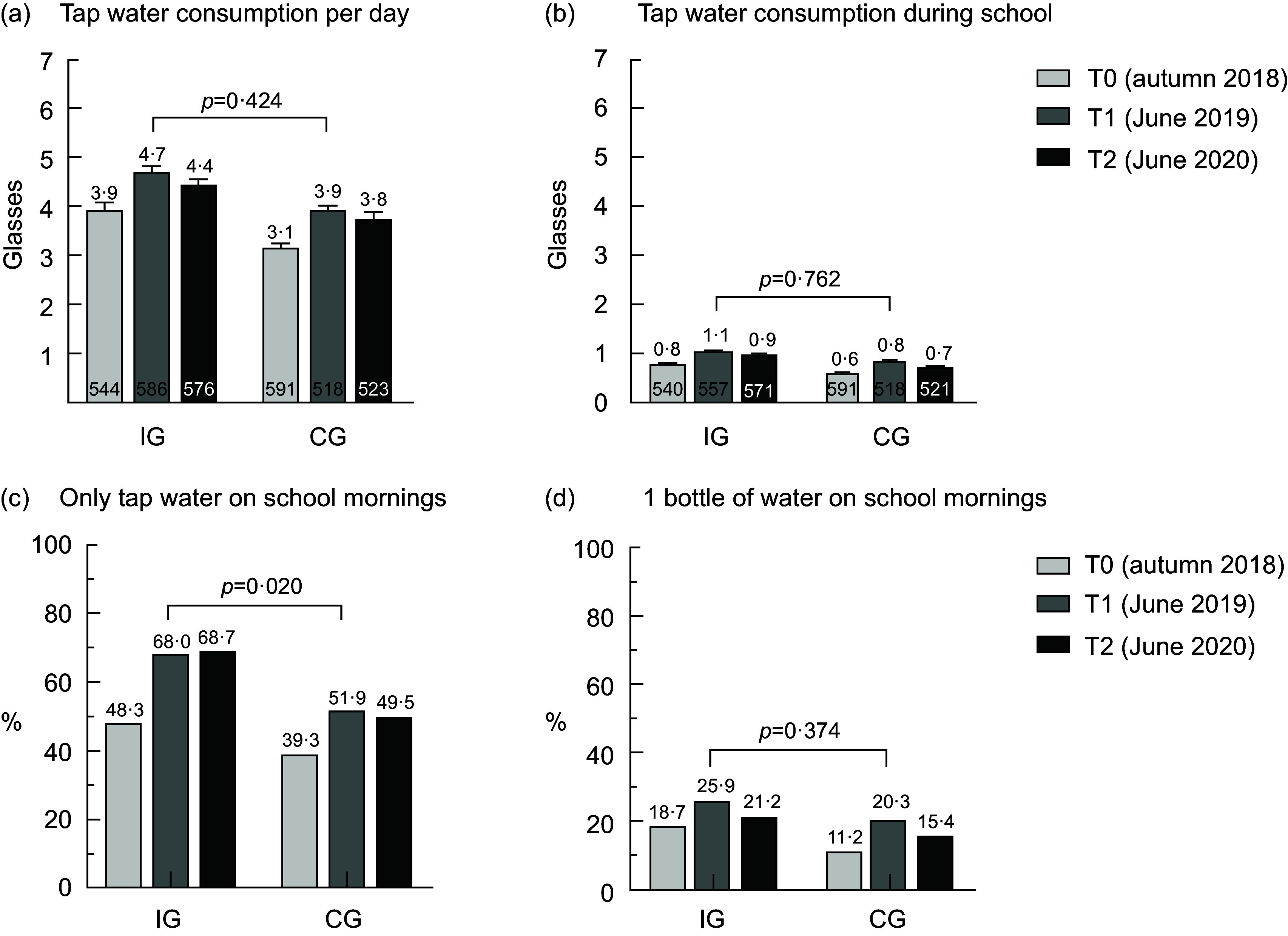
Beverage consumption among schoolchildren over time in the IG and CG. IG, intervention group; CG, control group; T0, baseline; T1, after the intervention at 9 months; T2, 1-year follow-up after the intervention; the numbers in the bottom of Fig. 2(a) and (b) denote the number of pupils; values are means and standard error of the mean; *P*-values for the difference in the time trends between the IG and CG account for the cluster effect on the class level

The consumption of tap water per d improved in both groups in a similar way: in the IG increasing from 3·9 ± 0·1 glasses to 4·7 ± 0·1 after the intervention (T1) and then decreasing again to 4·4 ± 0·1 after the 1-year follow-up (T2) and similarly in the CG increasing from 3·1 ± 0·1 glasses to 3·9 ± 0·1 at T1 and to 3·8 ± 0·1 at T2 (Fig. 2(a)). No statistically significant difference in the time trend between the IG and CG was found. A similar trend was seen for tap water consumption in the mornings (Fig. 2(b)) as well as for total water (tap and mineral) consumption per d (see online Supplemental file Fig. S2) and the consumption of tap water as well as total water relative to the total drinking volume (see online Supplemental file Figs. S7 and S8). Soft drink consumption per d was stable in both groups (see online Supplemental file Fig. S5), but juice consumption per d decreased in the IG compared to the CG (*P* = 0·053, see online Supplemental file Fig. S6).

The proportion of children who only drank tap water in the morning during school increased in the IG from 48·3 % at baseline to 68·0 % at T1 and 68·7 % at T2 and was statistically significantly different from the CG, where the proportion increased from 39·3 % at baseline to 51·9 % at T1 and 49·5 % at T2 (*P* = 0·020, Fig. 2(c)). The proportions of children who drank approximately one bottle of tap water during school mornings were generally low, with 18·7 % in the IG and 11·2 % in the CG at baseline. The changes over time were similar in both groups, with an increase at T1 and a decrease again at T2 (*P* = 0·374, Fig. 2(d)).

### Attitude of schoolchildren

The proportion of children who agreed to the statement ‘I like to drink water’ with either ‘Yes, right’ or ‘Yes, mostly’ was already high at baseline (IG: 86·3 %, CG: 89·3 %) and did not change much over time between the groups (*P* = 0·272, see online Supplemental file Table S2 and Fig. S9). Agreement with the statement ‘I like soft drinks’ changed over time and was lowest in the IG and higher in the CG immediately after the intervention at T1 (*P* = 0·065, see online Supplemental file Table S2 and Fig. S10). Similar results were found for juice and juice with water (*P* = 0·099, see online Supplemental file, Table S2 and Fig. S11). The expressed preference for tap water in school decreased in both groups over time but was more pronounced in the CG (*P* = 0·055, see online Supplemental file Table S2 and Fig. S13).

The responses for water (tap and mineral) as the preferred beverage when thirsty were similar in the IG and CG, and the increasing trend over time was similar in both groups (IG: 49·8, 53·5 and 54·8 %, CG: 48·8, 50·8 and 52·5 %, *P* = 0·935; see online Supplemental file Table S3).

### Process evaluation

At T1, 78·0 % (*n* 46) of teachers reported having drinking rules in the classroom, and more than 90 % established these rules because of the programme. At T2, fewer teachers stated that they had drinking rules in their classroom (58·6 %, *n* 41). Similar results were seen with the use of the drinking pass during class. At T1, 69·0 % (*n* 40) of teachers used the drinking pass during class, whereas at T2 only 30·0 % (*n* 21) did.

At T1, on average 82 % of children in a class used the programme drinking bottle (range from 17 to 100 %). At T2, this value decreased to 74 %, whereas at T2 more pupils used their own drinking bottles during class (T0: 24 % *v*. T1: 16 %), according to teachers’ reports. At T1, 95 % of teachers (*n* 56) reported that children used the programme drinking bottles every day. This value decreased to 84 % (*n* 59) at T2.

Furthermore, we also included questions for the pupils in the paper-and-pencil questionnaire regarding the implementation of the ‘H2NOE Water Schools’ programme, that is, the extent of the use of water bottles. At T1, 55·9 % of the pupils in the third grade (*n* 329/589) used the programme water bottle every day. This value decreased to 34·1 % (*n* 200/586) at T2 and was statistically significant (*P* < 0·001).

At T1, 83·6 % (*n* 51) of schoolteachers in the IG addressed the topic of water drinking during classes. At T2, the proportion sank to 62·2 % (*n* 46) of teachers who addressed the topic of water drinking in face-to-face lessons during regular class. As the second semester of the school year was, in large part, carried out through distance learning and the last 6 weeks of the school year through alternating face-to-face lessons for half the class due to the COVID-19 pandemic lockdown measures in place at that time in Austria, we also asked about the distance lessons, and only 14·9 % of teachers (*n* 11) in the IG addressed the topic of water drinking during distance learning in spring 2020.

**Fig. 3:**
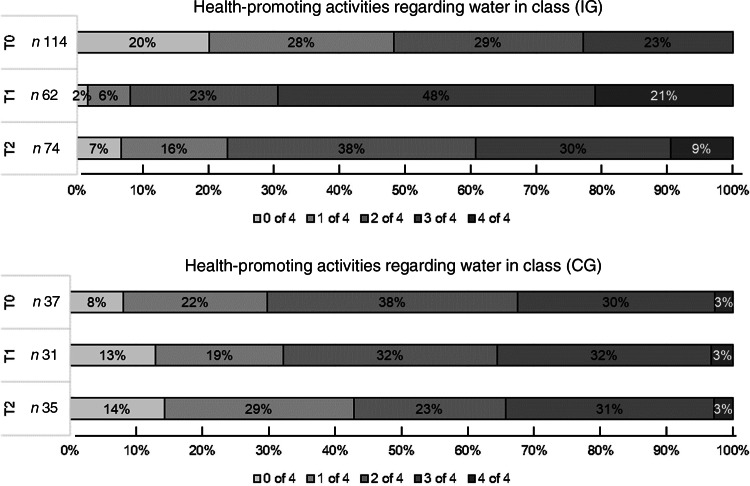
Number of health-promoting activities regarding water in class (%) in the IG and CG. IG, intervention group; CG, control group; T0, baseline; T1, after the intervention at 9 months; T2, 1-year follow-up after the intervention; n, number of teachers who answered the questionnaire; *P*-value < 0·001 for the difference in the time trends between the IG and CG

We also administered online questionnaires to teachers at the CG schools and asked them and the teachers at the IG schools about the health promotion activities in their own class at all three time points. The proportion of teachers reporting the implementation of all four of the listed health promotion activities regarding water remained stable in the CG over the three time points, at approximately 3 %, whereas in the IG at baseline, none of the teachers reported having installed all four of the listed health promotion activities regarding water in their class. This proportion reached 21·0 % in the IG at T1 and was 9·5 % at T2 (Fig. 3). At T2, the proportions were similar between the IG and CG (*P* < 0·001 for the overall difference in the time trend between the groups).

At the end of the first school year with the ‘H2NOE Water Schools’ programme (at T1), 61·9 % of the schoolchildren (*n* 365/590) in the IG liked the programme, 23·7 % (*n* 140/590) were neutral and 10·2 % disliked it (*n* 60/590); 41·0 % of the teachers graded the programme as very good (*n* 25/61), 55·7 % as good (*n* 34/61) and 3·3 % as satisfactory (*n* 2/61); 73·3 % of the headmasters graded the programme as very good (*n* 11/15) and 26·7 % as good (*n* 4/15). At T2, the pupils’ approval of the programme was somewhat lower but was still high, with 53·5 % (*n* 315/589) liking the programme and 30·4 % (*n* 179/589) being neutral. The teachers’ and headmasters’ ratings at T2 were similar to those at T1.

## Discussion

In this non-randomised controlled cluster trial studying the ‘H2NOE Water Schools in Lower Austria’ programme, we showed that children from both the IG and CG increased their consumption of tap water per d and during school mornings, but there was no difference between the groups. Hence, providing refillable water bottles and educational material did not increase the consumption of tap water. The consumption of soft drinks remained stable over all three measurements in both the IG and the CG, but the juice consumption decreased slightly in the IG right after the intervention. The main aims of the ‘H2NOE Water Schools in Lower Austria’ programme were that pupils consume approximately one bottle of water (500 ml) and that they exclusively drink tap water during their school day, which lasts until before lunch. The proportion of children who only drank tap water on school mornings increased significantly in the IG from baseline to T1 compared to the CG (*P* = 0·020), but there were no changes over time between the groups for the proportion of pupils who drank approximately one bottle of tap water during school mornings. The attitude of schoolchildren towards drinking water did not change much in either group. The liking of drinking tap water at school decreased over time in both groups but was more pronounced in the CG.

There are several points we want to raise to interpret these results. First, we speculate that the survey on drinking behaviour in the CG acted as a nudge for school headmasters and teachers to take on interventions to increase pupils’ water consumption during the school year. In fact, 72 % of the control schools conducted health-promoting activities regarding water consumption during the 2018–2019 school year. Although we did not ask schools to do so, such activities were not discouraged either. In conversations with headmasters, some even mentioned that the survey reminded them to again focus on healthy beverage intake in their school. From the data on health promotion activities in classrooms, we could infer that classes in the CG were quite motivated regarding health promotion, because the proportions of classes having three or four of the four listed health-promoting activities regarding water in their class were relatively stable in the CG, whereas the proportions were similar between the IG and CG only at T2. At baseline in the IG, none of the teachers reported having instituted all four of the listed health-promoting activities regarding water in their class, and this proportion reached 21 % at T1. Furthermore, it is important that the baseline water consumption was significantly higher in the IG, and that half the intervention schools reported that they conducted health-promoting activities regarding water consumption before becoming a ‘Water School’. A third important factor is the seasonality. The baseline measurements were conducted in autumn (September 2018); the post-intervention and follow-up measurements were taken in early summer (June 2019 and June 2020). The consumption of water may be higher in warmer weather^(44–46)^. Furthermore, due to logistical reasons, the control schools’ baseline questionnaires were sent approximately 2 weeks after the intervention schools’, and there may have already been colder weather. However, we cannot verify this because we did not ask pupils to include the date when filling in the questionnaire. Fourth, the results could be the true results, showing that the intervention did not have an effect.

Previous studies have shown that the active promotion of water drinking using the provision of cool, filtered water or cup dispensers near water fountains increased the consumption of water^(47–50)^. Further, the distribution of water bottles had a positive effect on water consumption^(36,37)^. Other studies showed that encouraging water consumption in place of SSB decreased the consumption of SSB^(51,52)^ and increased water consumption^(51,53,54)^. A study recently published by Smit *et al.* used influence agents from their own classroom to promote water consumption as an alternative to SSB consumption and showed less SSB consumption, but no difference was found in water consumption^(55)^.

In terms of process evaluation, 78·0 % of the teachers in the IG reported having drinking rules in the class at T1, and over 90 % established them because of the ‘Water Schools’ programme. After the 1-year follow-up (at T2), the proportion of classes with drinking rules decreased to 58·6 %. Similarly, the use of the programme bottle decreased from an average of 82 % to 74 % from T1 to T2. This is in agreement with the pupils’ statements, where the self-reported daily use of the programme bottle decreased from 55·9 % to 34·1 % from T1 to T2. Addressing the topic of water during class lessons also decreased during the follow-up from 83·6 % at T1 to 62·2 % at T2. However, the second semester of the school year was, in large part, carried out through distance learning and the last 6 weeks of the school year in alternating face-to-face lessons due to COVID-19 pandemic lockdown measures. Therefore, opportunities to address the topic of water during lessons were rare. Nevertheless, a small proportion of teachers (14·9 %) did address the topic of water during distance learning in spring 2020.

### Strengths and limitations

This study had a number of strengths. First, the sample size was large. Second, the follow-up time was long, and measurements were taken at three time points. Consequently, we can draw conclusions about the sustainability of the intervention. As far as we know, none of the previous studies of school interventions to increase water consumption had a follow-up of 1 year after the intervention. Even in the large German cluster-randomised controlled trial by Muckelbauer *et al*., only the water flow of the water fountains was measured at the 19-month follow-up, but no individual data on water consumption of schoolchildren^(56)^. In general, long-term studies on school health promotion are rare, and the sustainability of public health interventions after the end of the intervention is relatively unexplored in the school setting compared to the health sector^(57,58)^.

There are some limitations that are important when interpreting the results of our study. First, the assessment of children’s drinking behaviours was based on self-report using a validated 24-h recall questionnaire developed by Muckelbauer *et al.*^(42)^. Water intake may potentially be underestimated when using 24-h recall^(59)^. Furthermore, the measurement may have been inaccurate: First, because we adapted the 24-h recall questionnaire according to the authors’ recommendations (i.e. omitting the quantity category ‘empty glass’ and the category ‘other beverages’)^(42)^. By omitting the ‘other beverages’ category, it cannot be ruled out that children consumed more of a beverage category that was not recorded at all. Therefore, the quantification of the total beverage volume may be flawed, as Muckelbauer *et al.* also stated in their validation study^(42)^. Second, the pupil survey took place in the morning. We asked teachers to hand out the questionnaires during a lesson after the morning break at approximately 9:30 a.m. So pupils completed the questionnaires between 9:30 a.m. and noon. The results for the water consumption during the morning may differ depending on the time the questionnaire was completed. In some instances, only half the morning had passed, and we cannot extrapolate the results to the whole morning. The data on beverage consumption during the whole day are therefore better suited for comparison purposes. However, measuring the consumption of beverage intake in young children remains a challenge^(60)^.

Second, recruitment of the schools was based on a convenience sample. The intervention schools were contacted and invited for participation through our practice partner, who also created and financed the ‘H2NOE Water Schools in Lower Austria’ programme. They mainly contacted schools that had previously expressed interest or that had already taken part in other school health promotion interventions. The control schools, on the other hand, were contacted by us, and we contacted all eligible schools from three different districts in the north-eastern part of the federal state of Lower Austria.

Third, parental involvement in the intervention was minimal. Parents were informed of the intervention via parent letters. Parental modelling of certain eating and drinking behaviours has been shown to influence children’s diet, and the availability of SSB in the home has also been strongly associated with SSB consumption among children^(61)^. Hence, an approach to improve the effectiveness of the intervention could be to motivate parents to set an example at home for their children with regard to water drinking.

Fourth, the response rate of the teacher questionnaires ranged between 10 and 80 %. Therefore, it is not possible to draw conclusions about all teachers.

Fifth, the transferability to countries without high-quality tap water (as in Austria) may be limited.

## Conclusions

In conclusion, a simple environmental health promotion intervention providing free refillable water bottles and educational material may increase the water consumption of elementary schoolchildren. However, the impact of the intervention may not differ significantly from drawing school headmasters’ and teachers’ attention to focus on healthy beverages.

## Supplementary material

For supplementary material accompanying this paper visit https://doi.org/10.1017/S1368980021003438. click here to view supplementary material

## References

[1] Cruz F , Ramos E , Lopes C et al. (2018) Tracking of food and nutrient intake from adolescence into early adulthood. Nutrition 55, 84–90. 29980092 10.1016/j.nut.2018.02.015

[2] Cutler GJ , Flood A , Hannan P et al. (2009) Major patterns of dietary intake in adolescents and their stability over time. J Nutr 139, 323–328. 19091799 10.3945/jn.108.090928

[3] Frémeaux AE , Hosking J , Metcalf BS et al. (2011) Consistency of children’s dietary choices: annual repeat measures from 5 to 13 years (EarlyBird 49). Br J Nutr 106, 725–731. 21736842 10.1017/S0007114511000705

[4] Mikkilä V , Räsänen L , Raitakari O et al. (2005) Consistent dietary patterns identified from childhood to adulthood: the cardiovascular risk in Young Finns Study. Br J Nutr 93, 923–931. 16022763 10.1079/bjn20051418

[5] Movassagh EZ , Baxter-Jones AD , Kontulainen S et al. (2017) Tracking dietary patterns over 20 years from childhood through adolescence into young adulthood: the Saskatchewan Pediatric Bone Mineral Accrual Study. Nutrients 9, 990. 28885565 10.3390/nu9090990 PMC5622750

[6] Kelder SH , Perry CL , Klepp K-I et al. (1994) Longitudinal tracking of adolescent smoking, physical activity, and food choice behaviors. Am J Public Health 84, 1121–1126. 8017536 10.2105/ajph.84.7.1121 PMC1614729

[7] Nicklaus S , Boggio V , Chabanet C et al. (2004) A prospective study of food preferences in childhood. Food Qual Prefer 15, 805–818.

[8] Demory-Luce D , Morales M , Nicklas T et al. (2004) Changes in food group consumption patterns from childhood to young adulthood: the Bogalusa Heart Study. J Am Diet Assoc 104, 1684–1691. 15499355 10.1016/j.jada.2004.07.026

[9] Reedy J & Krebs-Smith SM (2010) Dietary sources of energy, solid fats, and added sugars among children and adolescents in the United States. J Am Diet Assoc 110, 1477–1484. 20869486 10.1016/j.jada.2010.07.010 PMC3428130

[10] Te Morenga L , Mallard S & Mann J (2013) Dietary sugars and body weight: systematic review and meta-analyses of randomised controlled trials and cohort studies. BMJ 346, e7492. 10.1136/bmj.e7492 23321486

[11] Vartanian LR , Schwartz MB & Brownell KD (2007) Effects of soft drink consumption on nutrition and health: a systematic review and meta-analysis. Am J Public Health 97, 667–675. 17329656 10.2105/AJPH.2005.083782 PMC1829363

[12] Malik VS , Pan A , Willett WC et al. (2013) Sugar-sweetened beverages and weight gain in children and adults: a systematic review and meta-analysis. Am J Clin Nutr 98, 1084–1102. 23966427 10.3945/ajcn.113.058362 PMC3778861

[13] Malik VS , Popkin BM , Bray GA et al. (2010) Sugar-sweetened beverages and risk of metabolic syndrome and type 2 diabetes: a meta-analysis. Diabetes Care 33, 2477–2483. 20693348 10.2337/dc10-1079 PMC2963518

[14] Malik VS & Hu FB (2019) Sugar-sweetened beverages and cardiometabolic health: an update of the evidence. Nutrients 11, 1840. 31398911 10.3390/nu11081840 PMC6723421

[15] Moynihan P & Petersen PE (2004) Diet, nutrition and the prevention of dental diseases. Public Health Nutr 7, 201–226. 14972061 10.1079/phn2003589

[16] Moynihan PJ & Kelly SA (2014) Effect on caries of restricting sugars intake: systematic review to inform WHO guidelines. J Dent Res 93, 8–18. 24323509 10.1177/0022034513508954 PMC3872848

[17] Sheiham A & James WPT (2014) A new understanding of the relationship between sugars, dental caries and fluoride use: implications for limits on sugars consumption. Public Health Nutr 17, 2176–2184. 24892213 10.1017/S136898001400113X PMC10282617

[18] World Health Organization (2003) Diet, Nutrition, and the Prevention of Chronic Diseases: Report of a Joint WHO/FAO Expert Consultation Series 916. Geneva: World Health Organization. 12768890

[19] World Health Organization (2015) Guideline: Sugars Intake for Adults and Children. no. 978 92 4 154902 8. Geneva: World Health Organization. 25905159

[20] Popkin BM , D’Anci KE & Rosenberg IH (2010) Water, hydration, and health. Nutr Rev 68, 439–458. 20646222 10.1111/j.1753-4887.2010.00304.x PMC2908954

[21] Fadda R , Rapinett G , Grathwohl D et al. (2012) Effects of drinking supplementary water at school on cognitive performance in children. Appetite 59, 730–737. 22841529 10.1016/j.appet.2012.07.005

[22] Bottin JH , Morin C , Guelinckx I et al. (2019) Hydration in children: what do we know and why does it matter? Ann Nutr Metab 74, 11–18. 10.1159/000500340 31203294

[23] Kaushik A , Mullee MA , Bryant TN et al. (2007) A study of the association between children’s access to drinking water in primary schools and their fluid intake: can water be ‘cool’ in school? Child Care Health Dev 33, 409–415. 17584396 10.1111/j.1365-2214.2006.00721.x

[24] Edmonds CJ & Burford D (2009) Should children drink more water? The effects of drinking water on cognition in children. Appetite 52, 776–779. 19501780 10.1016/j.appet.2009.02.010

[25] Booth P , Taylor B & Edmonds C (2012) Water supplementation improves visual attention and fine motor skills in schoolchildren. Educ Health 30, 75–79.

[26] Booth P (2015) The effect of water consumption on school children’s fine motor skills, cognitive function and mood. PhD Thesis, University of East London.

[27] EFSA Panel on Dietetic Products, Nutrition, and Allergies (2010) Scientific opinion on dietary reference values for water. EFSA J 8, 1459.

[28] Deutsche Gesellschaft für Ernährung (DGE), Österreichische Gesellschaft für Ernährung (ÖGE) & Schweizerische Gesellschaft für Ernährung (SGE) (editors) (2015) D-A-CH-Referenzwerte Für Die Nährstoffzufuhr (Reference Values for Nutrient Intake for Germany, Austria and Switzerland (D-A-CH)). Frankfurt am Main: Umschau Braus GmbH.

[29] Vieux F , Maillot M , Rehm CD et al. (2020) Trends in tap and bottled water consumption among children and adults in the United States: analyses of NHANES 2011–16 data. Nutr J 19, 10. 31996207 10.1186/s12937-020-0523-6 PMC6990513

[30] Vieux F , Maillot M , Constant F et al. (2016) Water and beverage consumption among children aged 4–13 years in France: analyses of INCA 2 (Étude Individuelle Nationale des Consommations Alimentaires 2006–2007) data. Public Health Nutr 19, 2305–2314. 26878881 10.1017/S1368980015003614 PMC4981897

[31] Guelinckx I , Iglesia I , Bottin JH et al. (2015) Intake of water and beverages of children and adolescents in 13 countries. Eur J Nutr 54, Suppl. 2, 69–79. 26072216 10.1007/s00394-015-0955-5 PMC4473084

[32] World Health Organization (2018) Global Standards for Health Promoting Schools. Geneva, Switzerland: World Health Organization.

[33] Bevans KB , Sanchez B , Teneralli R et al. (2011) Children’s eating behavior: the importance of nutrition standards for foods in schools. J Sch Health 81, 424–429. 21668883 10.1111/j.1746-1561.2011.00611.x PMC3196854

[34] Marteau TM , Hollands GJ & Fletcher PC (2012) Changing human behavior to prevent disease: the importance of targeting automatic processes. Science 337, 1492–1495. 22997327 10.1126/science.1226918

[35] Patel AI & Schmidt LA (2020) Healthy beverage initiatives in higher education: an untapped strategy for health promotion. Public Health Nutr 24, 136–138. 33087201 10.1017/S1368980020003766 PMC10195485

[36] Elder JP , Holub CK , Arredondo EM et al. (2014) Promotion of water consumption in elementary school children in San Diego, USA and Tlaltizapan, Mexico. Salud Publica Mex 56, Suppl. 2, s148–s156. 25629247 10.21149/spm.v56s2.5179

[37] Smit CR , de Leeuw RNH , Bevelander KE et al. (2016) A social network-based intervention stimulating peer influence on children’s self-reported water consumption: a randomized control trial. Appetite 103, 294–301. 27085637 10.1016/j.appet.2016.04.011

[38] van de Gaar VM , Jansen W , van Grieken A et al. (2014) Effects of an intervention aimed at reducing the intake of sugar-sweetened beverages in primary school children: a controlled trial. Int J Behav Nutr Phys Act 11, 98. 25060113 10.1186/s12966-014-0098-8 PMC4222660

[39] Muckelbauer R , Libuda L , Clausen K et al. (2009) Promotion and provision of drinking water in schools for overweight prevention: randomized, controlled cluster trial. Pediatrics 123, e661–667. 19336356 10.1542/peds.2008-2186

[40] Moore GF , Audrey S , Barker M et al. (2015) Process evaluation of complex interventions: medical research council guidance. BMJ 350, h1258. 25791983 10.1136/bmj.h1258 PMC4366184

[41] W.K. Kellogg Foundation (2004) Logic Model Development Guide. Using Logic Models to Bring Together Planning, Evaluation, and Action. Michigan, USA: W.K. Kellogg Foundation.

[42] Muckelbauer R , Libuda L & Kersting M (2009) Relative validity of a self-completion 24 h recall questionnaire to assess beverage consumption among schoolchildren aged 7 to 9 years. Public Health Nutr 13, 187–195. 19650966 10.1017/S1368980009990759

[43] Collins D (2003) Pretesting survey instruments: an overview of cognitive methods. Qual Life Res 12, 229–238. 12769135 10.1023/a:1023254226592

[44] Beltrán-Aguilar ED , Barker L , Sohn W et al. (2015) Water intake by outdoor temperature among children aged 1–10 years: implications for community water fluoridation in the US. Public Health Rep 130, 362–371. 26346578 10.1177/003335491513000415 PMC4547571

[45] Dimkić D (2020) Temperature impact on drinking water consumption. Environ Sci Proc 2, 31.

[46] Galagan DJ , Vermillion JR , Nevitt GA et al. (1957) Climate and fluid intake. Public Health Rep 72, 484. 13432123 PMC2031312

[47] Kenney EL , Gortmaker SL , Carter JE et al. (2015) Grab a cup, fill it up! An intervention to promote the convenience of drinking water and increase student water consumption during school lunch. Am J Public Health 105, 1777–1783. 26180950 10.2105/AJPH.2015.302645 PMC4539814

[48] Loughridge JL & Barratt J (2005) Does the provision of cooled filtered water in secondary school cafeterias increase water drinking and decrease the purchase of soft drinks? J Hum Nutr Diet 18, 281–286. 16011564 10.1111/j.1365-277X.2005.00622.x

[49] Patel AI , Bogart LM , Elliott MN et al. (2011) Increasing the availability and consumption of drinking water in middle schools: a pilot study. Prev Chronic Dis 8, A60. 21477500 PMC3103565

[50] Patel AI , Grummon AH , Hampton KE et al. (2016) A trial of the efficacy and cost of water delivery systems in San Francisco Bay Area middle schools, 2013. Prev Chronic Dis 13, E88. 27390074 10.5888/pcd13.160108 PMC4951080

[51] Abi Haidar G & Afifi R (2011) Jarrib Baleha – a pilot nutrition intervention to increase water intake and decrease soft drink consumption among school children in Beirut. J Med Liban 59, 55–64. 21834488

[52] Sichieri R , Trotte AP , de Souza RA et al. (2008) School randomised trial on prevention of excessive weight gain by discouraging students from drinking sodas. Public Health Nutr 12, 197–202. 18559131 10.1017/S1368980008002644

[53] Avery A , Bostock L & McCullough F (2015) A systematic review investigating interventions that can help reduce consumption of sugar-sweetened beverages in children leading to changes in body fatness. J Hum Nutr Diet 28, 52–64. 25233843 10.1111/jhn.12267 PMC4309175

[54] Siega-Riz AM , El Ghormli L , Mobley C et al. (2011) The effects of the HEALTHY study intervention on middle school student dietary intakes. Int J Behav Nutr Phys Act 8, 7. 21294869 10.1186/1479-5868-8-7 PMC3041997

[55] Smit CR , de Leeuw RN , Bevelander KE et al. (2021) Promoting water consumption among children: a three-arm cluster randomized controlled trial testing a social network intervention. Public Health Nutr 24, 2324–2336. 33243308 10.1017/S1368980020004802 PMC8145454

[56] Muckelbauer R , Libuda L , Clausen K et al. (2009) Long-term process evaluation of a school-based programme for overweight prevention. Child Care Health Dev 35, 851–857. 19702638 10.1111/j.1365-2214.2009.00993.x

[57] Herlitz L , MacIntyre H , Osborn T et al. (2020) The sustainability of public health interventions in schools: a systematic review. Implement Sci 15, 1–28. 31906983 10.1186/s13012-019-0961-8 PMC6945701

[58] Langford R , Bonell C , Jones H et al. (2015) The World Health Organization’s Health Promoting Schools framework: a Cochrane systematic review and meta-analysis. BMC Public Health 15, 1–15. 25886385 10.1186/s12889-015-1360-y PMC4339015

[59] Bardosono S , Monrozier R , Permadhi I et al. (2015) Total fluid intake assessed with a 7-d fluid record *v.* a 24-h dietary recall: a crossover study in Indonesian adolescents and adults. Eur J Nutr 54, 17–25. 26072215 10.1007/s00394-015-0954-6 PMC4473025

[60] Warren J , Guelinckx I , Livingstone B et al. (2018) Challenges in the assessment of total fluid intake in children and adolescents: a discussion paper. Eur J Nutr 57, 43–51. 29923117 10.1007/s00394-018-1745-7 PMC6008368

[61] Story M , Kaphingst KM , Robinson-O’Brien R et al. (2008) Creating healthy food and eating environments: policy and environmental approaches. Annu Rev Public Health 29, 253–272. 18031223 10.1146/annurev.publhealth.29.020907.090926

