# Supplementary material for: Evaluation of the ‘H2NOE Water Schools’ programme to promote water consumption in elementary schoolchildren: a non-randomised controlled cluster trial
Source: Public Health Nutr. 2021 Aug 13;25(1):159–69. doi: 10.1017/S1368980021003438 (PMC8825982; doi:10.1017/S1368980021003438)
Supplement: Supplementary file 1 [file S1368980021003438sup001.docx]

# **Supplementary material** for the article “Evaluation of the ‘H2NOE Water Schools’ programme to promote water consumption in elementary school children – a non-randomised controlled cluster trial”

# Methods

## Food consumption of schoolchildren

Further secondary outcomes were the changes in food consumption of pupils: consumption of fruit and berries; vegetables (raw or cooked); sweets, chocolate or ice-cream; soft drinks (lemonade, cola and iced tea); fast food (French fries, chicken nuggets, pizza, McDonald's, sausages); salty snacks (crisps, popcorn, salted pretzel sticks). We used a modified version of the validated food frequency questionnaire from the Health Behaviour in School-aged Children (HBSC) study 2013/2014^(1)^ and simplified the response categories based on the results from our pilot test, because the age of responding children in our study with approximately 8 years was lower than in the HBSC study with 11-15 years. We calculated a nutrition score to categorize the diet of the children into a health-promoting diet, moderately healthy diet and not health-promoting diet. The nutrition score refers only to selected items that should either be consumed in any case in a health-promoting diet (vegetables, fruit) or only in small quantities (fatty, sweet, salty) or should be avoided altogether (soft drinks and caffeinated beverages). The nutrition score is based on the Austrian Dietary recommendations for children aged 4 to 10 years^(2)^.

The item to measure change in breakfast consumption (i.e. frequency of eating breakfast during school days and at the weekend) was also based on the corresponding validated item in the HBSC study^(1)^, but with simplified response categories based on the results from our pilot test.

# Results

## Beverage consumption of schoolchildren

Table S1: Mean and SE, with pupils as units of analysis and taking into account the cluster effect at class level, of beverage consumption, at baseline (T0), post-test (T1) and follow-up (T2) by study group [pupil questionnaire]

|  | **IG** | | | | | | **CG** | | | | | | **P-value** |
| --- | --- | --- | --- | --- | --- | --- | --- | --- | --- | --- | --- | --- | --- |
|  | **T0 (n=540)** | | **T1 (n=557)** | | **T2 (n=571)** | | **T0 (n=591)** | | **T1 (n=518)** | | **T2 (n=521)** | |  |
|  | **Mean** | **SE** | **Mean** | **SE** | **Mean** | **SE** | **Mean** | **SE** | **Mean** | **SE** | **Mean** | **SE** |  |
| **Beverage consumption in glasses** |  |  |  |  |  |  |  |  |  |  |  |  |  |
| Tap water per day | 3.9 | 0.1 | 4.7 | 0.1 | 4.4 | 0.1 | 3.1 | 0.1 | 3.9 | 0.1 | 3.8 | 0.1 | 0.424 |
| Tap water during school | 0.8 | 0.0 | 1.1 | 0.0 | 0.9 | 0.0 | 0.6 | 0.0 | 0.8 | 0.0 | 0.7 | 0.0 | 0.762 |
| Mineral water per day | 1.0 | 0.1 | 1.0 | 0.1 | 1.0 | 0.1 | 1.2 | 0.1 | 1.4 | 0.1 | 1.3 | 0.1 | 0.625 |
| Water (tap and mineral) per day | 4.9 | 0.1 | 5.7 | 0.1 | 5.5 | 0.1 | 4.3 | 0.1 | 5.3 | 0.1 | 5.1 | 0.1 | 0.286 |
| Tea per day | 0.3 | 0.0 | 0.2 | 0.0 | 0.3 | 0.0 | 0.4 | 0.0 | 0.2 | 0.0 | 0.3 | 0.0 | 0.507 |
| Milk and chocolate milk per day | 0.8 | 0.0 | 0.7 | 0.0 | 0.5 | 0.0 | 0.7 | 0.0 | 0.6 | 0.0 | 0.5 | 0.0 | 0.955 |
| Soft drinks (lemonade, cola, iced tea) per day | 0.6 | 0.1 | 0.7 | 0.1 | 0.7 | 0.1 | 0.5 | 0.0 | 0.7 | 0.1 | 0.7 | 0.1 | 0.248 |
| Juice and juice with water per day | 1.8 | 0.1 | 1.5 | 0.1 | 1.6 | 0.1 | 1.7 | 0.1 | 1.8 | 0.1 | 1.8 | 0.1 | 0.053 |
| **Water consumption relative to total drinking volume in %** |  |  |  |  |  |  |  |  |  |  |  |  |  |
| Tap water relative to total drinking volume (%) | 47.9 | 1.4 | 53.6 | 1.2 | 52.3 | 1.3 | 40.9 | 1.3 | 45.2 | 1.4 | 45.7 | 1.5 | 0.786 |
| Total water (tap and mineral) relative to total drinking volume (%) | 58.8 | 1.3 | 64.4 | 1.2 | 63.7 | 1.1 | 55.5 | 1.2 | 61.1 | 1.2 | 61.7 | 1.3 | 0.737 |
|  | **%** | **-** | **%** | **-** | **%** | **-** | **%** | **-** | **%** | **-** | **%** | **-** |  |
| Proportion of children who only drink tap water in the morning during school (%) | 48.3 | - | 68.0 | - | 68.7 | - | 39.3 | - | 51.9 | - | 49.5 | - | 0.020 |
| Proportion of children who drank approximately one bottle of tap water during the school mornings (%) | 18.7 | - | 25.9 | - | 21.2 | - | 11.2 | - | 20.3 | - | 15.4 | - | 0.374 |


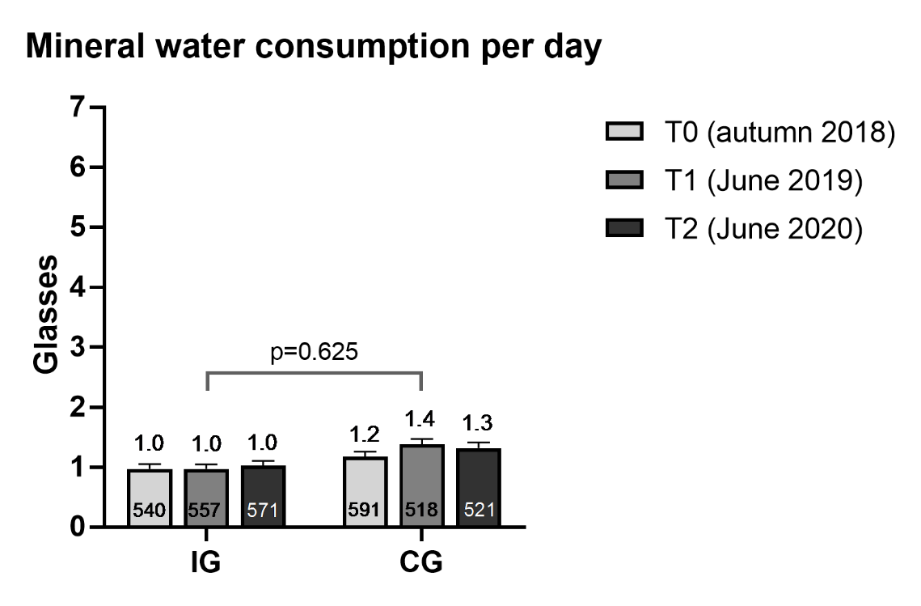


Figure S1: Mineral water consumption per day among school children over time in IG and CG.

IG, intervention group; CG, control group; T0, baseline; T1, after the intervention at 9 months; T2, one year follow-up after the intervention; the numbers in the bottom of the Figure denote the number of pupils; values are means ± SE (standard error of the mean); p-values for the difference in time trends between IG and CG taking into account the cluster effect on the class level.


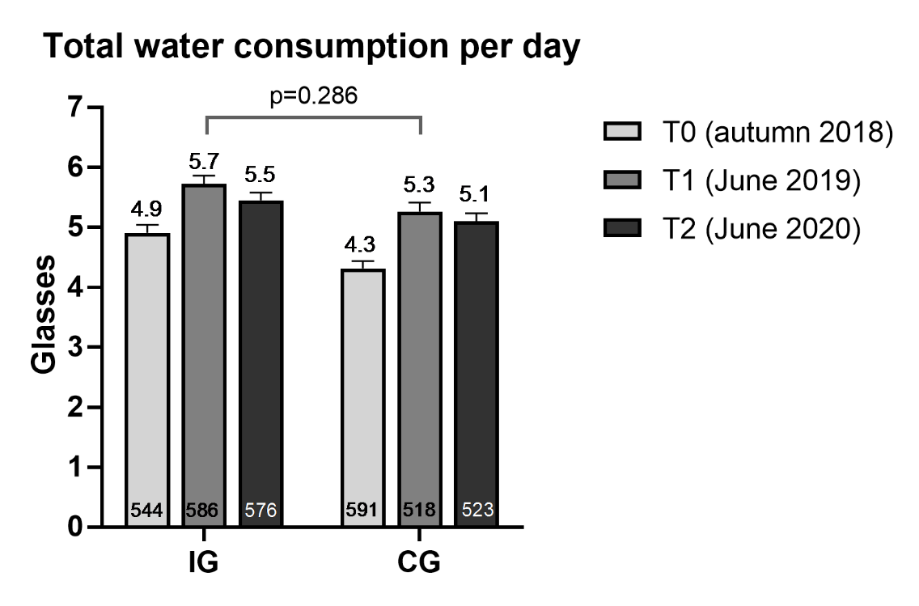


Figure S2: Total water consumption per day among school children over time in IG and CG.

IG, intervention group; CG, control group; T0, baseline; T1, after the intervention at 9 months; T2, one year follow-up after the intervention; the numbers in the bottom of the Figure denote the number of pupils; values are means ± SE (standard error of the mean); p-values for the difference in time trends between IG and CG taking into account the cluster effect on the class level.


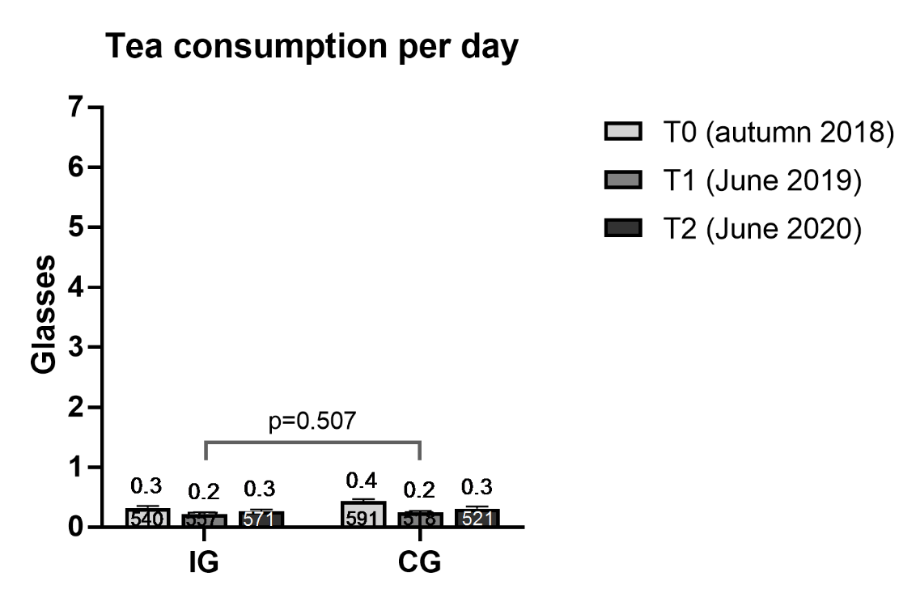


Figure S3: Tea consumption per day among school children over time in IG and CG.

IG, intervention group; CG, control group; T0, baseline; T1, after the intervention at 9 months; T2, one year follow-up after the intervention; the numbers in the bottom of the Figure denote the number of pupils; values are means ± SE (standard error of the mean); p-values for the difference in time trends between IG and CG taking into account the cluster effect on the class level.


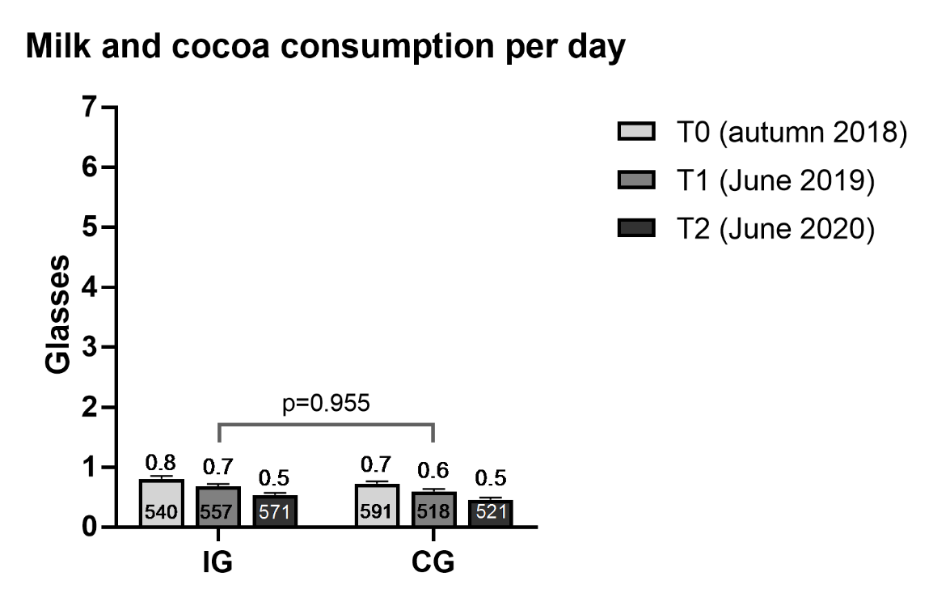


Figure S4: Milk and chocolate milk consumption per day among school children over time in IG and CG.

IG, intervention group; CG, control group; T0, baseline; T1, after the intervention at 9 months; T2, one year follow-up after the intervention; the numbers in the bottom of the Figure denote the number of pupils; values are means ± SE (standard error of the mean); p-values for the difference in time trends between IG and CG taking into account the cluster effect on the class level.


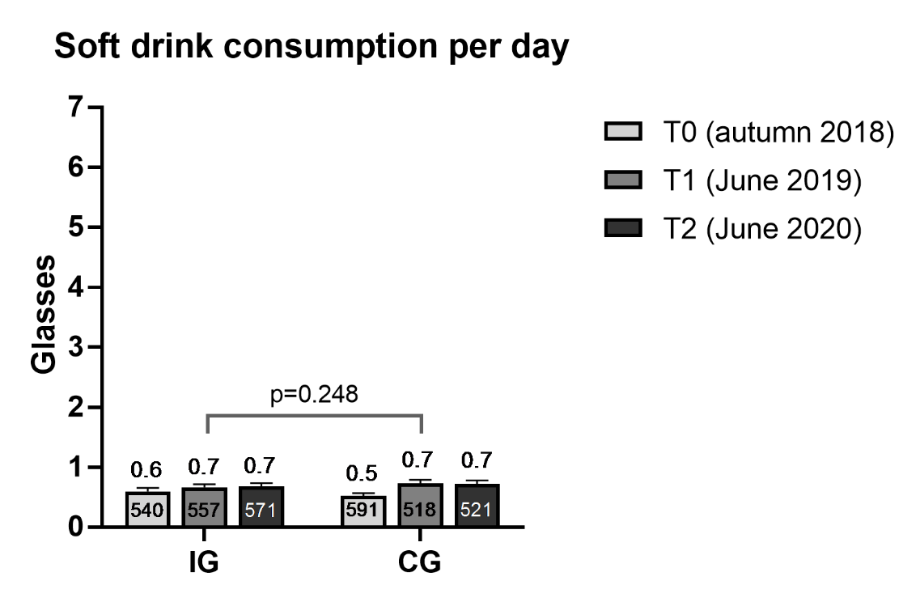


Figure S5: Soft drink consumption per day among school children over time in IG and CG.

IG, intervention group; CG, control group; T0, baseline; T1, after the intervention at 9 months; T2, one year follow-up after the intervention; the numbers in the bottom of the Figure denote the number of pupils; values are means ± SE (standard error of the mean); p-values for the difference in time trends between IG and CG taking into account the cluster effect on the class level.


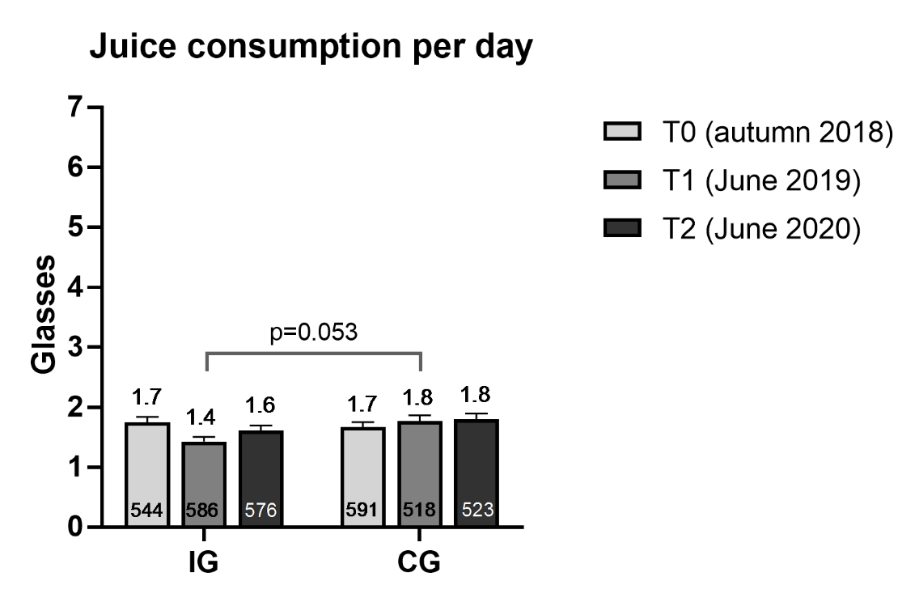


Figure S6: Juice and juice with water consumption per day among school children over time in IG and CG.

IG, intervention group; CG, control group; T0, baseline; T1, after the intervention at 9 months; T2, one year follow-up after the intervention; the numbers in the bottom of the Figure denote the number of pupils; values are means ± SE (standard error of the mean); p-values for the difference in time trends between IG and CG taking into account the cluster effect on the class level.


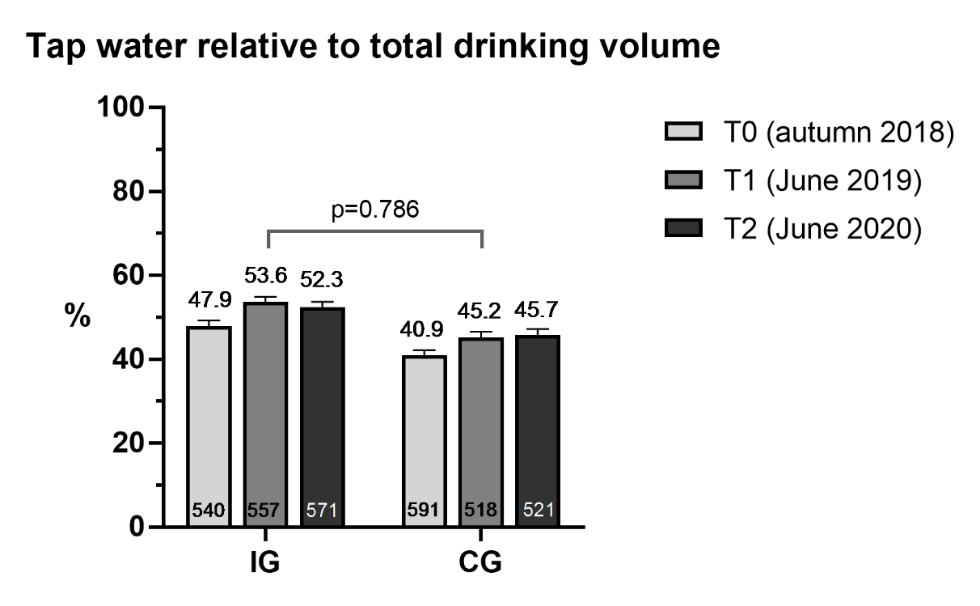


Figure S7: Consumption of tap water relative to total drinking volume (%) among school children over time in IG and CG.

IG, intervention group; CG, control group; T0, baseline; T1, after the intervention at 9 months; T2, one year follow-up after the intervention; the numbers in the bottom of the Figure denote the number of pupils; values are means ± SE (standard error of the mean); p-values for the difference in time trends between IG and CG taking into account the cluster effect on the class level.


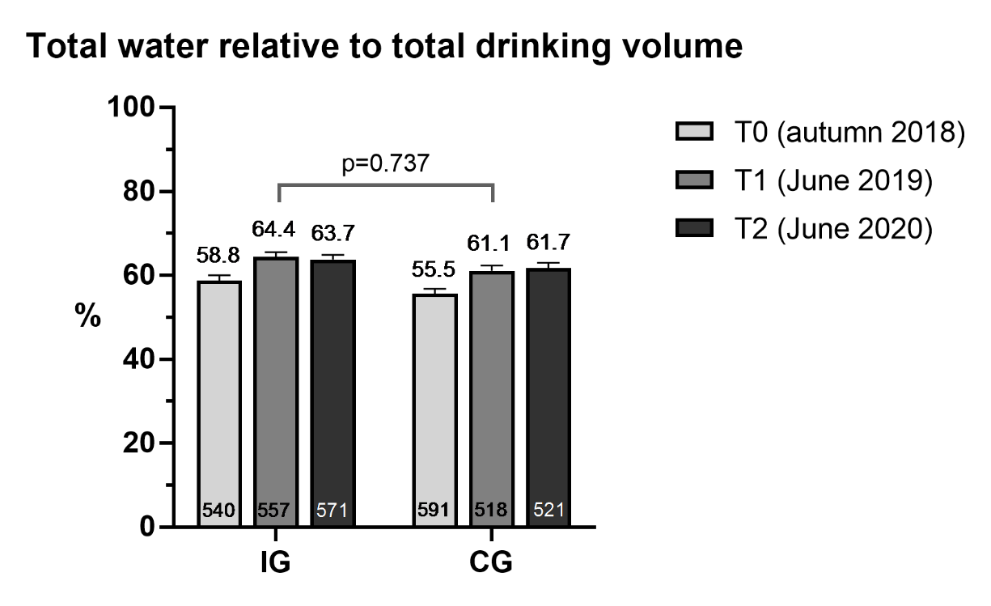


Figure S8: Consumption of total water relative to total drinking volume (%) among school children over time in IG and CG.

IG, intervention group; CG, control group; T0, baseline; T1, after the intervention at 9 months; T2, one year follow-up after the intervention; the numbers in the bottom of the Figure denote the number of pupils; values are means ± SE (standard error of the mean); p-values for the difference in time trends between IG and CG taking into account the cluster effect on the class level.

## Attitude of schoolchildren

Table S2: Preference for water and SSBs, 5-point Likert scale recoded by collapsing the response categories “yes, that's right” and “yes, mostly” to “yes” as well as “no, mostly not” and “no, not true” to “no”; valid percent for “yes” [pupil questionnaire]

|  | **IG** | | | **CG** | | | **P-value** |
| --- | --- | --- | --- | --- | --- | --- | --- |
| **%** | **T0** | **T1** | **T2** | **T0** | **T1** | **T2** |  |
| I like to drink water. | 86.3 | 88.5 | 87.4 | 89.3 | 87.9 | 88.8 | 0.272 |
| I like to drink lemonade, cola, or iced tea. | 54.7 | 50.3 | 56.0 | 52.1 | 54.8 | 53.7 | 0.065 |
| I like to drink juice or juice with water. | 76.6 | 71.9 | 73.1 | 75.6 | 76.2 | 71.4 | 0.099 |
| I like tap water. | 81.3 | 82.8 | 84.8 | 78.8 | 81.5 | 81.5 | 0.731 |
| I like the tap water at school. | 72.8 | 70.0 | 66.0 | 70.8 | 65.4 | 54.4 | 0.055 |

Generalized Linear Models, p-values for differences in time trend between IG and CG, with adjustment for clustering according to classroom


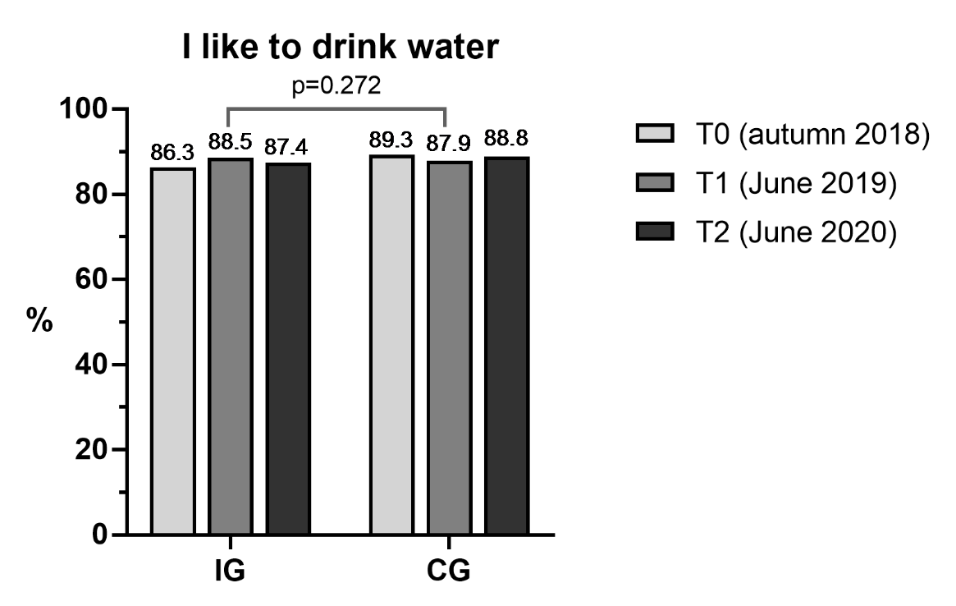


Figure S9: Agreement to the statement “I like to drink water” among school children (%) over time in IG and CG.

IG, intervention group; CG, control group; T0, baseline; T1, after the intervention at 9 months; T2, one year follow-up after the intervention; p-values for the difference in time trends between IG and CG taking into account the cluster effect on the class level.


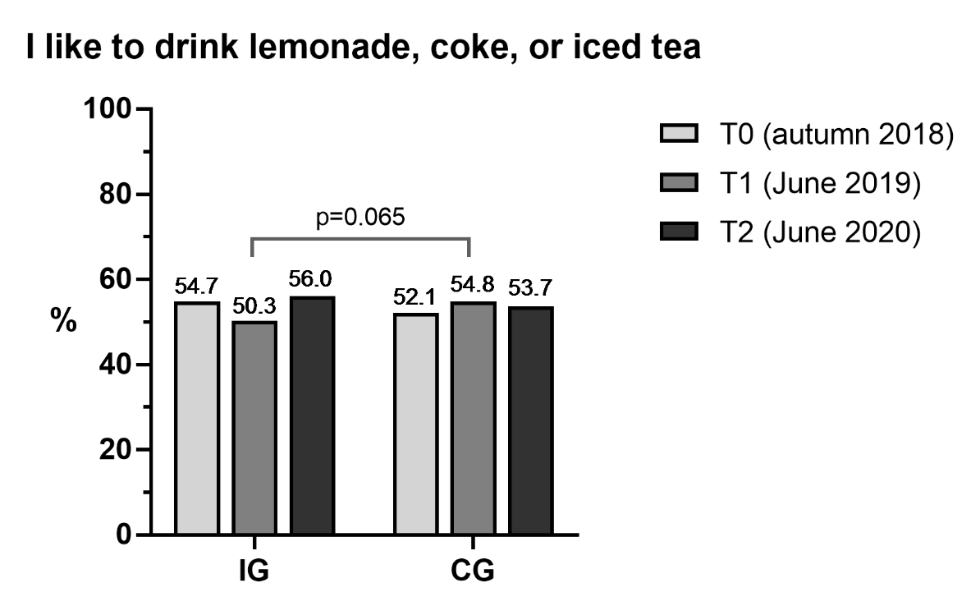


Figure S10: Agreement to the statement “I like to drink lemonade, cola, or iced tea” among school children (%) over time in IG and CG.

IG, intervention group; CG, control group; T0, baseline; T1, after the intervention at 9 months; T2, one year follow-up after the intervention; p-values for the difference in time trends between IG and CG taking into account the cluster effect on the class level.


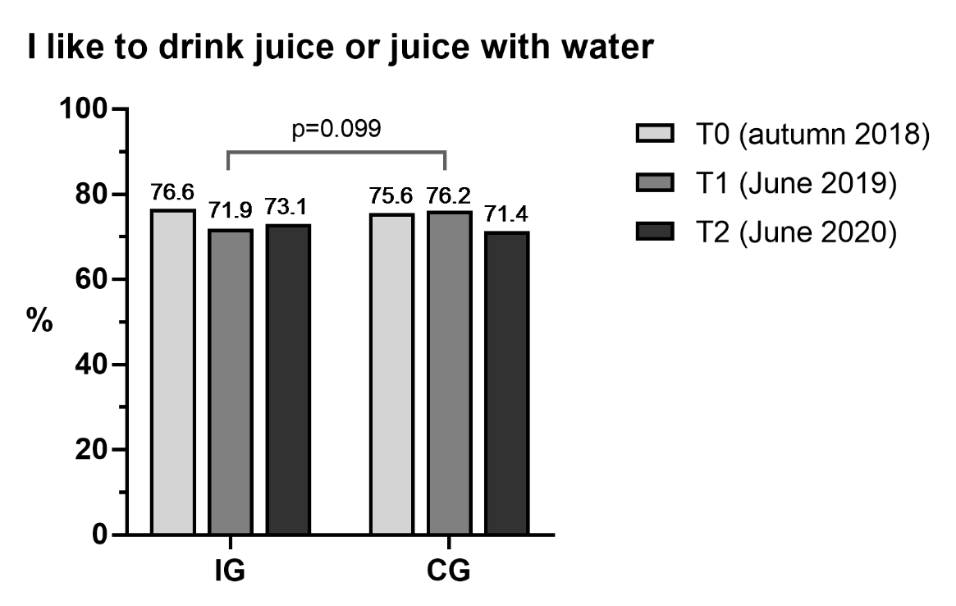


Figure S11: Agreement to the statement “I like to drink juice or juice with water” among school children (%) over time in IG and CG.

IG, intervention group; CG, control group; T0, baseline; T1, after the intervention at 9 months; T2, one year follow-up after the intervention; p-values for the difference in time trends between IG and CG taking into account the cluster effect on the class level.


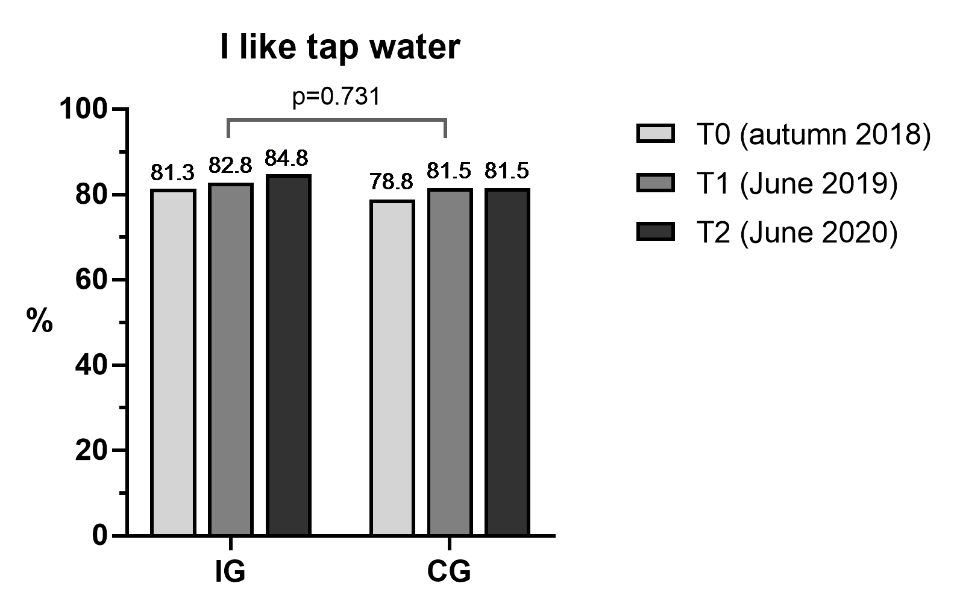


Figure S12: Agreement to the statement “I like tap water” among school children (%) over time in IG and CG.

IG, intervention group; CG, control group; T0, baseline; T1, after the intervention at 9 months; T2, one year follow-up after the intervention; p-values for the difference in time trends between IG and CG taking into account the cluster effect on the class level.


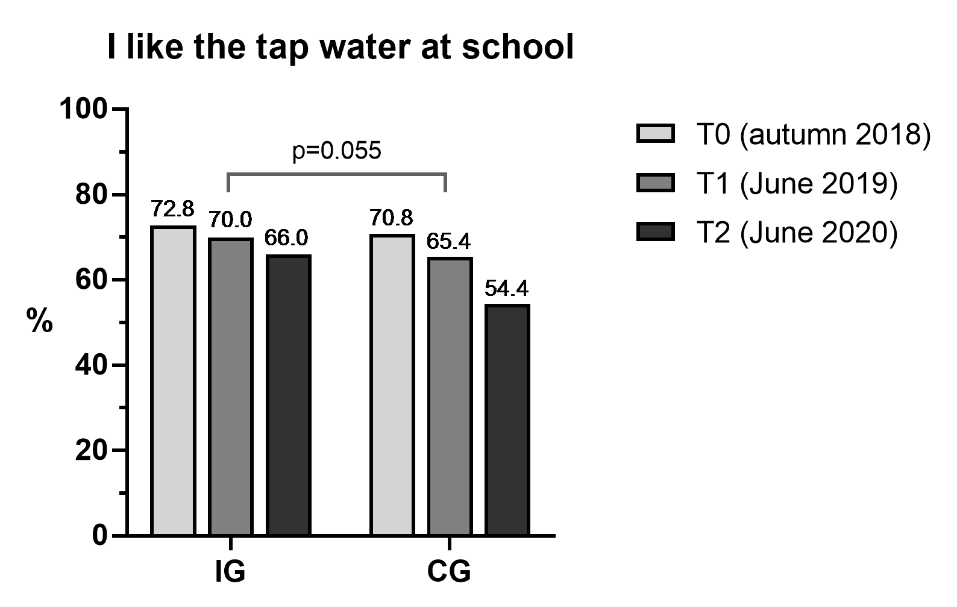


Figure S13: Agreement to the statement “I like the tap water at school” among school children (%) over time in IG and CG.

IG, intervention group; CG, control group; T0, baseline; T1, after the intervention at 9 months; T2, one year follow-up after the intervention; p-values for the difference in time trends between IG and CG taking into account the cluster effect on the class level.

Table S3: Favourite beverage when thirsty, valid percent [pupil questionnaire]

|  | **IG** | | | **CG** | | | **P-value** |
| --- | --- | --- | --- | --- | --- | --- | --- |
| **%** | **T0** | **T1** | **T2** | **T0** | **T1** | **T2** | 0.935 |
| Tap water | 34.2 | 36.2 | 38.0 | 31.1 | 28.4 | 32.0 |  |
| Mineral water | 15.6 | 17.3 | 16.8 | 17.7 | 22.4 | 20.5 |  |
| Soft drinks (lemonade, cola, iced tea) | 29.1 | 24.7 | 24.0 | 26.0 | 26.2 | 24.7 |  |
| Juice or juice with water | 19.3 | 18.8 | 18.6 | 23.0 | 19.3 | 20.1 |  |
| Tea | 0.6 | 0.2 | 1.1 | 0.4 | 0.2 | 0.6 |  |
| Milk | 0.2 | 0.0 | 0.0 | 0.2 | 0.2 | 0.4 |  |
| Chocolate milk | 0.2 | 0.8 | 0.0 | 0.9 | 0.2 | 0.0 |  |
| Vanilla milk | 0.0 | 0.0 | 0.0 | 0.2 | 0.0 | 0.0 |  |
| Sports drinks | 0.2 | 0.2 | 0.2 | 0.2 | 0.0 | 0.2 |  |
| Flavoured water | 0.0 | 0.6 | 0.7 |  | 0.4 | 1.2 |  |
| Others | 0.6 | 1.1 | 0.6 | 0.4 | 2.4 | 0.2 |  |

Generalized Linear Model, p-values for differences in time trend between IG and CG, with adjustment for clustering according to classroom

## Food consumption of schoolchildren

Table S4: Nutrition score, categorized, valid percent [pupil questionnaire]

|  | **IG** | | | **CG** | | | **P-value** |
| --- | --- | --- | --- | --- | --- | --- | --- |
| **%** | **T0** | **T1** | **T2** | **T0** | **T1** | **T2** | 0.002 |
| Health-promoting diet | 8.6 | 11.7 | 15.5 | 10.3 | 8.3 | 11.9 |  |
| Moderately healthy diet | 55.5 | 61.0 | 60.5 | 57.6 | 59.8 | 61.2 |  |
| Not health-promoting diet | 35.9 | 27.3 | 24.0 | 32.1 | 31.9 | 26.9 |  |

Generalized Linear Model, p-values for differences in time trend between IG and CG, with adjustment for clustering according to classroom

## Breakfast consumption of schoolchildren

Table S5: Breakfast consumption, valid percent [pupil questionnaire]

|  | **IG** | | | **CG** | | | **P-value** |
| --- | --- | --- | --- | --- | --- | --- | --- |
| **%** | **T0** | **T1** | **T2** | **T0** | **T1** | **T2** |  |
| I never eat breakfast when there is school. | 16.2 | 18.0 | 21.0 | 20.5 | 23.1 | 23.6 | 0.947 |
| I sometimes have breakfast when there is school. | 18.2 | 18.7 | 18.3 | 19.7 | 19.7 | 22.1 |  |
| I usually have breakfast when there is school. | 9.7 | 11.4 | 9.8 | 10.2 | 11.1 | 12.9 |  |
| I always have breakfast when there is school. | 55.9 | 51.9 | 50.8 | 49.6 | 46.1 | 41.5 |  |
| I never eat breakfast at the weekend. | 4.8 | 3.9 | 3.4 | 2.4 | 5.8 | 5.2 | 0.076 |
| I sometimes have breakfast at the weekend. | 10.4 | 12.2 | 10.3 | 16.9 | 17.3 | 15.3 |  |
| I usually have breakfast at the weekend. | 15.1 | 13.7 | 19.1 | 12.8 | 14.3 | 21.0 |  |
| I always have breakfast at the weekend. | 69.7 | 70.2 | 67.2 | 68.0 | 62.7 | 58.5 |  |

Generalized Linear Models, p-values for differences in time trend between IG and CG, with adjustment for clustering according to classroom

## Process evaluation (implementation dose)

Table S6: Extent of water bottle use (programme bottle), valid percent, IG only [pupil questionnaire]

| **%** | **T1 (n=589)** | **T2 (n=596)** |
| --- | --- | --- |
| Never or almost never | 15.6 | 35.5 |
| Sometimes | 26.0 | 28.2 |
| Every day | 55.9 | 34.1 |
| I do not know | 2.5 | 2.2 |

Table S7: Extent of water bottle use, IG only [teacher questionnaire]

| **%** | **T1 (n=59)** | **T2 (n=70)** |
| --- | --- | --- |
| Programme bottle | 82 (range 17-100) | 74 (range 0-100) |
| Own drinking bottle | 16 (range 0-80) | 24 (range 0-100) |

Table S8: Drinking rules in class, valid percent, IG only [teacher questionnaire]

| **%** | **T1 (n=59)** | **T2 (n=70)** |
| --- | --- | --- |
| Yes | 78.0 | 58.6 |
| No | 22.0 | 28.6 |
| Only at the beginning of the school year 2019/2020 | Not part of the questionnaire | 12.9 |

Table S9: Use of drinking pass, valid percent, IG only [teacher questionnaire]

| % | **T1 (n=58)** | **T2 (n=70)** |
| --- | --- | --- |
| Yes | 69.0 | 30.0 |
| No | 31.0 | 70.0 |

Table S10: Addressing the topic water during regular lessons, valid percent, IG only [teacher questionnaire]

| **%** | **T1 (n=61)** | **T2 (n=74)** |
| --- | --- | --- |
| Yes (during face-to-face classes) | 83.6 | 62.2 |
| Yes (during home schooling) | Not part of the questionnaire | 14.9 |

Table S11: Mean number of posters displayed at class and school, IG only [headmaster questionnaire]

|  | **T1 (n=20)** |
| --- | --- |
| Mean number of posters displayed per class | 6 (range 0-13) |
| Mean number of posters displayed per school | 3 (range 0-13) |

## Process evaluation (implementation reach)

Parents were informed about the programme in 16 out of 20 intervention schools (mean number of parents letters was 2) [headmaster questionnaire].

In 92% of classrooms, drinking rules were implemented during the first year of the programme (range 50-100%) and all classes in the third grade implemented drinking rules [headmaster questionnaire].

In 60% of intervention schools, information about the water schools programme was displayed on the school homepage and further 25% planned to provide information about the programme on their homepage [headmaster questionnaire].

## Health promoting activities in class

Table S12: Number of health promoting activities regarding water in class, valid percent [teacher questionnaire]

|  | **IG** | | | **CG** | | | **P-value** |
| --- | --- | --- | --- | --- | --- | --- | --- |
| **%** | **T0 (n=114)** | **T1 (n=62)** | **T2 (n=74)** | **T0 (n=37)** | **T1 (n=31)** | **T2 (n=35)** | <0.001 |
| 0 of 4 activities | 20.2% | 1.6% | 6.8% | 8.1% | 12.9% | 14.3% |  |
| 1 of 4 activities | 28.1% | 6.5% | 16.2% | 21.6% | 19.4% | 28.6% |  |
| 2 of 4 activities | 28.9% | 22.6% | 37.8% | 37.8% | 32.3% | 22.9% |  |
| 3 of 4 activities | 22.8% | 48.4% | 29.7% | 29.7% | 32.3% | 31.4% |  |
| 4 of 4 activities | 0.0% | 21.0% | 9.5% | 2.7% | 3.2% | 2.9% |  |

# References

1. Currie C, Inchley J, Molcho M *et al.* (2014) Health behaviour in school-aged children (HBSC) study protocol: background, methodology and mandatory items for the 2013/14 survey.

2. Bruckmüller MU, Dieminger-Schnürch B, Hesina S *et al.* (2017) *Richtig essen von Anfang an!* . Ernährungsempfehlungen für Kinder im Alter von 4 bis 10 Jahren. Wien: Österreichische Agentur für Gesundheit und Ernährungssicherheit GmbH (AGES).
